# Association between serum cotinine levels and cognitive function in Americans aged 60 and older: A cross-sectional study

**DOI:** 10.1097/MD.0000000000048784

**Published:** 2026-05-22

**Authors:** Jieqiong Zhang, Guanxun Zhang, Ciai Yan, Guangyu Cheng, Zhixin Sun, Weiping Cheng

**Affiliations:** a Department of First Clinical School, Heilongjiang University of Chinese Medicine, Harbin, Heilongjiang Province, PR China; b Department of Research Centre of Translational Medicine of Traditional Chinese Medicine, The First Affiliated Hospital of Heilongjiang University of Chinese Medicine, Harbin, Heilongjiang Province, PR China; c Department of Preventive Treatment Centre, The First Affiliated Hospital of Heilongjiang University of Chinese Medicine, Harbin, PR China; d Acupuncture Department II, The First Affiliated Hospital of Heilongjiang University of Chinese Medicine, Harbin, Heilongjiang Province, PR China.

**Keywords:** cognitive function, cotinine, nicotine, serum biomarkers

## Abstract

Cognitive decline poses a major public health burden on the aging population, necessitating the identification of modifiable risk factors. Cotinine, a key nicotine metabolite and an established biomarker of tobacco exposure, has demonstrated biologically plausible associations with cognitive function. This study examined the association between serum cotinine levels and cognitive function in US adults aged ≥60 years. We conducted a cross-sectional study including 2781 participants aged ≥60 years, using data from the United States National Health and Nutrition Examination Survey from 2011 to 2014. Sampling weights were applied in all analyses to account for the complex survey design and ensure national representativeness. We used multivariable linear regression supplemented by stratified analyses, sensitivity analyses, and restricted cubic splines to model nonlinear associations. Multivariable-adjusted analyses showed a significant inverse association between serum cotinine levels and cognitive function (β = −0.85, 95% confidence interval: −1.62 to −0.08, *P* = .034). Subgroup analyses consistently demonstrated this inverse association across strata (all *P* for interaction > .05). Sensitivity analyses supported the robustness of these findings. Curve fitting revealed an L-shaped relationship (inflection point: log_2_cotinine = −0.16; 95% confidence interval: −0.30 to −0.03, *P* for nonlinearity = .022), with a significant inverse association observed below this threshold (*P* = .017), whereas the association plateaued above the threshold (*P* = .063), suggesting a potential threshold-dependent association between serum cotinine and cognitive function. This cross-sectional study found a significant inverse association between serum cotinine levels and cognitive function in older adults in the United States, which was observed as an L-shaped relationship with a threshold of log_2_cotinine = −0.16. These findings contribute to evidence on tobacco exposure and cognitive outcomes in aging populations.

## 1. Introduction

Cognitive function underpins the capacity to perform activities of daily living, sustain social engagement, and achieve occupational goals. With an aging global population, the rising burden of cognitive impairment and dementia necessitates urgent public health interventions. The Global Burden of Disease study (2019) estimates that 152.8 million people will be diagnosed with dementia globally by 2050,^[1]^ imposing substantial pressure on healthcare systems and socioeconomic resources.

Multiple modifiable and non-modifiable factors, including age, education, health status, and lifestyle, underlie variations in cognitive function. The association between nicotine and cognitive function is of considerable scientific interest. Nicotine is mainly derived from tobacco and nicotine replacement therapy products, and exhibits substantial variations in concentration and absorption methods,^[2]^ which may be related to dependence and cognitive deterioration. which may be related to dependence and cognitive deterioration. Serum cotinine is validated as a biomarker of nicotine exposure owing to its pharmacokinetic stability and prolonged half-life.^[3]^ Cotinine is the principal metabolite of nicotine that potentially impairs cognitive function through dysregulated cholinergic signaling and altered hippocampal neurocircuitry, suggesting that it is a modifiable risk factor associated with cognitive function.

Although nicotine has been reported to impair cognitive function, evidence indicates that cotinine is also associated with the inhibition of Alzheimer disease (AD) symptoms in specific pathological contexts and may represent a potential therapeutic intervention for cognitive disorders.^[4,5]^ However, systematic research quantifying the association between serum cotinine concentrations and cognitive function among adults aged ≥60 years in the United States remains limited. We explored this association using data from a nationally representative survey in the United States. Our findings highlight the association between serum cotinine and cognitive function, adding to what is known about these patterns at the population level. These findings may inform clinical research on cognitive decline interventions and offer epidemiological insights for developing preventive strategies.

## 2. Methods

### 2.1. Data source and study participants

**Figure 1.**
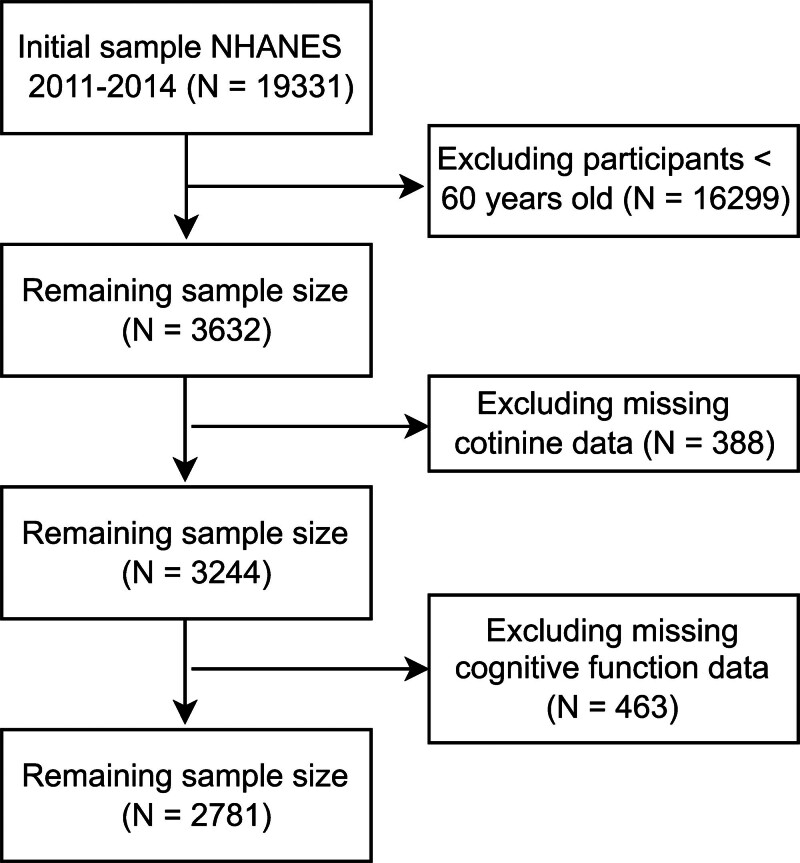
Inclusion and exclusion flowchart.

The National Health and Nutrition Examination Survey (hereinafter referred to as the survey) uses a complex, multistage stratified design to generate nationally representative estimates of health risks and nutritional status in the US population. Conducted biennially, the survey integrates household interviews with mobile examination center (MEC) assessments. In this study, we analyzed cycles from 2011 to 2014, during which the cognitive function questionnaire was exclusively administered to adults aged ≥60 years. Therefore, the analysis was restricted to this age group. As shown in Figure 1, from an initial sample of 19,331 participants, individuals aged <60 years, those with missing serum cotinine data, and those with incomplete cognitive function assessments were excluded from the analysis, yielding a final analytical sample size of 2781 individuals. To ensure population representativeness, we followed standard analytical guidelines for complex sample surveys and applied 2-year MEC weights (WTMEC2YR) calculated as 1/2 × WTMEC2YR in all analyses.^[6]^

### 2.2. Ethical approval and informed consent

This study used data from a continuous cross-sectional survey conducted by the Centers for Disease Control and Prevention and the National Center for Health Statistics. The survey protocol was approved by the National Center for Health Statistics Research Ethics Review Board, and written informed consent was obtained from all participants. Detailed documentation regarding ethical oversight is available from the Centers for Disease Control Ethics Review Board.^[7]^ The present analysis was deemed exempt from additional ethical review as it used de-identified publicly available data. This study adhered to the Strengthening the Reporting of Observational Studies in Epidemiology statement guidelines.

### 2.3. Outcome measures: cognitive function scores

Cognitive function was assessed using 3 standardized tests: the Animal Fluency Test (AFT), in which the participants named animals within 1 minute (score = total valid responses); the Digit Symbol Substitution Test (DSST), adapted from the Wechsler Adult Intelligence Scale, which was used to measure processing speed. Participants were asked to match symbols to numbers using a reference key and complete 133 items within 2 minutes (score = correct matches); and the CERAD Word Learning Subtest (CERAD W-L) was used to assess immediate and delayed verbal memory. Participants read 10 unrelated words aloud, followed by 3 immediate recall trials with randomized sequences (maximum 10/trial). Delayed recall was assessed after completing the other cognitive tests (AFT and DSST, 8–10 minutes post-encoding). A composite *z*-score was constructed by summing the standardized scores of all 3 tests, providing a comprehensive metric validated in population-based studies.^[8]^

### 2.4. Exposure: serum cotinine levels

Cotinine, the primary metabolite of nicotine, has a serum half-life of 15 to 20 hours, which is substantially longer than that of nicotine (0.5–3 hours), making it a sensitive biomarker for nicotine exposure. Although detectable in the serum, urine, and saliva, serum cotinine was used in the survey as a measure of nicotine exposure. Serum cotinine concentrations were obtained from the survey data, with measurement units consistent across survey cycles (ng/mL); data comparability was verified by the Centers for Disease Control and Prevention.^[9]^ Per established thresholds, serum cotinine levels <0.05 ng/mL were classified as indicating no tobacco exposure for demographic purposes.^[10,11]^

### 2.5. Covariates

Covariates were selected based on demographic characteristics, lifestyle behaviors, and chronic conditions, including age, sex, race/ethnicity, education, partner situation, poverty-income ratio (PIR), sedentary time, general health status, body mass index (BMI), smoking status, alcohol consumption, sleep disorders, depression, hypertension, heart disease, diabetes, and stroke. Continuous variables were age, BMI, and sedentary time, whereas all other variables were categorical. Race/ethnicity included non-Hispanic White, non-Hispanic Black, Mexican American, other Hispanic, and multiracial ethnicities. Education was grouped into 3 levels: middle school or below (<9th grade), high school education (9th–11th grade, including 12th grade without a diploma, high school graduate/general educational development, or equivalent), and university education or above (college or association of arts degree, college graduate, or above). Partner situations were categorized as living with a partner (married or living with a partner) or living alone (widowed, divorced, separated, or never married). Household income was categorized by the PIR as low (PIR ≤ 1.3), medium (1.3 < PIR ≤ 3.5), and high (PIR > 3.5). Hypertension was defined as meeting any of the following criteria: self-reported diagnosis of hypertension, hypertension diagnosed by a healthcare professional, average blood pressure of ≥140/90 mm Hg over 4 readings, or self-reported use of antihypertensive medication. Heart disease was defined as self-reported congestive heart failure, coronary heart disease, angina, or a heart attack. Smoking status was categorized based on lifetime cigarette consumption: nonsmokers (<100 lifetime cigarettes), active smokers (those who smoked daily or occasionally), and former smokers (those who had quit smoking entirely), with active and former smokers combined as eversmokers. Alcohol consumption was dichotomized as follows: those who reported having at least 12 drinks per year were classified as consumers, and those who did not were nonconsumers. Depressive symptoms were evaluated using the 9-item Patient Health Questionnaire, with a score of ≥10 indicating depression. Sleep disorders, diabetes, and stroke were identified through self-reported diagnoses by a physician.

For the primary analysis, covariates were selected for multivariable regression models based on any of the following criteria: univariate association with cognitive function (*P* < .01; Table S1, Supplemental Digital Content); alteration of the primary exposure coefficient by >10% when added to the model; and established clinical relevance, consistent with methodological guidelines.^[12–14]^ The primary models were adjusted for age, sex, race/ethnicity, PIR, partner situation, education, sedentary time, general health status, BMI, alcohol consumption, depression, stroke, hypertension, diabetes, and heart disease.

### 2.6. Statistical analyses

Categorical variables are presented as unweighted frequencies with weighted percentages and continuous variables as mean ± standard deviation or median (interquartile range). Missing covariate data (<20%) were handled using multivariate single imputation, with a Bayesian Ridge model used as the estimator in a round-robin imputation procedure.^[15]^ For baseline description, serum cotinine was dichotomized as <0.05 ng/mL and ≥0.05 ng/mL. Weighted multivariate linear regression models were used to examine the association between serum cotinine levels and cognitive function. Cotinine was analyzed as continuous untransformed values (μg/mL) and log_2_-transformed values, as well as categorically (<0.05 vs ≥0.05 ng/mL).

Covariates were sequentially incorporated into 6 models: crude model (unadjusted); model 1 was adjusted for age, sex, and race/ethnicity; model 2 was additionally adjusted for PIR, partner situation, and education; model 3 was further adjusted for sedentary time, general health status, BMI, and alcohol consumption; model 4 was additionally adjusted for depression, stroke, and hypertension; and model 5 was additionally adjusted for diabetes and heart disease, which was considered the primary model. Model 6 was further adjusted for smoking status. The primary analysis included models 1 to 5.

We conducted 2 sensitivity analyses to assess the robustness of our findings. First, we adjusted model 6 to examine the association between serum cotinine and cognitive function by applying this model to both the imputed (N = 2781) and complete-case (N = 2429) datasets. Second, we performed a complete-case analysis by reestimating all the models (crude through model 5) for the complete-case analysis (N = 2429). Results are presented as β coefficients with 95% confidence intervals (CIs).

Subgroup analyses assessed heterogeneity according to sex, education, partner situation, PIR, smoking status, depression, stroke, diabetes, and heart disease, using model 5. Interactions were evaluated using likelihood ratio tests, and the results were visualized in forest plots. Nonlinear associations between serum cotinine and cognitive function were characterized using restricted cubic splines with 3 knots (10th, 50th, and 90th percentiles) of log_2_cotinine.^[16]^ The *P* values for nonlinearity were obtained from the multivariable regression model. Segmented regression was used to identify the inflection points at which the slope of the association was steepest.^[17]^ Fitted curves were visualized.

All analyses incorporated MEC weights for national representativeness. Statistical analyses were performed using R software (version 4.2.2; http://www.R-project.org, The R Foundation) and Free Statistics software (version 2.4.0; FreeClinical Medical Technology Co., Ltd., Beijing, China).^[18]^ Statistical significance was determined at a two-tailed *P* < .05.

## 3. Results

### 3.1. Baseline characteristics of the study population

Table 1 presents the baseline characteristics of the 2781 study participants stratified by serum cotinine levels (<0.05 ng/mL: N = 1842; ≥0.05 ng/mL: N = 939). Significant differences were observed between the exposure groups across multiple variables. Participants with higher cotinine (≥0.05 ng/mL) were younger (median 67 vs 69 years, *P* < .001), more frequently male (53.00% vs 43.43%, *P* = .001), and disproportionately non-Hispanic Black (14.66% vs 5.39%, *P* < .001). Socioeconomic disparities were evident, with the higher cotinine group exhibiting lower educational attainment (university education: 48.64% vs 67.00%), reduced prevalence of living with a partner (57.53% vs 67.96%), and lower poverty-income ratios (PIR ≤ 1.3: 27.75% vs 14.05%) (all *P* < .001. Health behavior differences included higher active smoking (39.80% vs 0.05%, *P* < .001) and alcohol consumption (77.99% vs 70.88%, *P* = .002) in the high cotinine group. Participants with high cotinine exposure reported poorer self-rated health (fair/poor status: 28.85% vs 15.93%, *P* < .001) and higher comorbidities, such as heart disease (24.42% vs 15.29%, *P* < .001) and stroke history (8.53% vs 5.72%, *P* = .009). Cognitive function scores were lower in participants with higher serum cotinine levels (median 0.50 vs 1.24, *P* < .001). No statistically significant differences were observed in the survey cycle, sedentary time, BMI, sleep disorders, depression, hypertension, or diabetes prevalence (all *P* ≥ .05).

**Table 1:** Baseline characteristics of the study population.

Variables	Total (N = 2781)	Serum cotinine (ng/mL)		*P*
		<0.05 (N= 1842)	≥0.05 (N = 939)	
Cycle yr				.912
2011–2012	1266 (46.48)	826 (46.33)	440 (46.88)	
2013–2014	1515 (53.52)	1016 (53.67)	499 (53.12)	
Age (yr)	68 (63, 75)	69 (64, 76)	67 (63, 72)	<.001
Sex				.001
Male	1364 (46.12)	829 (43.43)	535 (53.00)	
Female	1417 (53.88)	1013 (56.57)	404 (47.00)	
Race/ethnicity				<.001
Non-Hispanic White	1358 (80.19)	995 (83.54)	363 (71.58)	
Non-Hispanic Black	634 (7.99)	299 (5.39)	335 (14.66)	
Mexican American	245 (3.36)	168 (3.3)	77 (3.52)	
Other Hispanic or Multi-Racial	544 (8.46)	380 (7.77)	164 (10.23)	
Education				<.001
Middle school education or below	312 (5.64)	181 (4.44)	131 (8.71)	
High school education	1046 (32.51)	617 (28.56)	429 (42.65)	
University education or above	1423 (61.85)	1044 (67.00)	379 (48.64)	
Partner situation				<.001
Live with a partner	1614 (65.04)	1142 (67.96)	472 (57.53)	
Live alone	1167 (34.96)	700 (32.04)	467 (42.47)	
PIR				<.001
≤1.3	833 (17.90)	450 (14.05)	383 (27.75)	
1.3–3.5	1063 (38.03)	691 (35.84)	372 (43.66)	
>3.5	885 (44.07)	701 (50.11)	184 (28.59)	
Sedentary time (h)	6 (4, 8)	6 (4, 8)	6.0 (4, 9)	.238
General health status				<.001
Good	2005 (80.44)	1402 (84.07)	603 (71.15)	
Fair	647 (16.48)	372 (13.78)	275 (23.40)	
Poor	129 (3.08)	68 (2.15)	61 (5.45)	
BMI (kg/m^2^)	29.03 ± 6.25	28.86 ± 5.82	29.46 ± 7.22	.234
Smoking status				<.001
Nonsmoking	1370 (49.73)	1103 (58.65)	267 (26.88)	
Former smoker	1051 (39.06)	737 (41.30)	314 (33.32)	
Active smoker	360 (11.20)	2 (0.05)	358 (39.80)	
Alcohol consumption				.002
No	876 (27.13)	648 (29.12)	228 (22.01)	
Yes	1905 (72.87)	1194 (70.88)	711 (77.99)	
Sleep disorders				.050
No	2449 (87.98)	1621 (88.91)	828 (85.60)	
Yes	332 (12.02)	221 (11.09)	111 (14.40)	
Depression				.088
No	2516 (92.48%)	1695 (93.49%)	821 (89.87%)	
Yes	265 (7.52%)	147 (6.51%)	118 (10.13%)	
Hypertension				.968
No	795 (32.74)	535 (32.77)	260 (32.67)	
Yes	1986 (67.26)	1307 (67.23)	679 (67.33)	
Heart disease				<.001
No	2290 (82.15)	1554 (84.71)	736 (75.58)	
Yes	491 (17.85)	288 (15.29)	203 (24.42)	
Diabetes				.103
No	2138 (80.79)	1435 (82.01)	703 (77.68)	
Yes	643 (19.21)	407 (17.99)	236 (22.32)	
Stroke				.009
No	2588 (93.49)	1731 (94.28)	857 (91.47)	
Yes	193 (6.51)	111 (5.72)	82 (8.53)	
Standardized cognitive function score	1.03 (−0.69, 2.60)	1.24 (−0.44, 2.81)	0.50 (−1.10, 2.02)	<.001

Weighted analyses were applied to all estimates. Continuous variables are summarized as weighted mean ± SD or weighted median (IQR); categorical variables as unweighted frequency (weighted percentage).

BMI = body mass index, IQR = interquartile range, PIR = poverty-income ratio, SD = standard deviation.

### 3.2. Multivariate linear regression analysis

Table 2 presents sample-weighted, multivariable linear regression results for the association between serum cotinine levels and cognitive function. For continuous cotinine scores, each 1 μg/mL increase in serum cotinine was consistently associated with lower cognitive function scores, although the magnitude of the association attenuated substantially with sequential covariate inclusion. In the crude model, the unadjusted β was −1.66, 95% CI: −2.64 to −0.69, *P* = .002. In model 1, adjustment for demographic factors (age, sex, and race/ethnicity) yielded a β of −2.29, 95% CI: −3.26 to −1.33; *P* < .001, which was numerically larger in magnitude. However, adjustment for socioeconomic factors (model 2: PIR, partner situation, and education) markedly attenuated the association (β = −0.89, 95% CI: −1.60 to −0.17, *P* = .018), representing a 46.4% reduction in the β coefficient compared with the unadjusted model. Further adjustments showed little additional change: lifestyle factors (model 3: sedentary time, general health status, BMI, and alcohol consumption; β = −0.80, 95% CI: −1.57 to −0.02, *P* = .044) and comorbidities (model 4: stroke, depression, and hypertension; β = −0.84, 95% CI: −1.58 to −0.10, *P* = .030). The primary model (model 5: additionally including heart disease and diabetes) showed a persistent significant inverse association (β = −0.85, 95% CI: −1.62 to −0.08, *P* = .034). For categorical cotinine exposure (≥0.05 ng/mL vs reference: <0.05 ng/mL), elevated levels were consistently associated with lower cognitive function scores across all models. The crude model showed −0.69 points lower scores (95% CI: −1.01 to −0.37; *P* < .001). After adjustments for demographics (model 1), this difference was −0.72 points (95% CI: −0.98 to −0.46; *P* < .001). Subsequent socioeconomic adjustment (model 2) attenuated the difference to −0.29 points (95% CI: −0.50 to −0.09, *P* = .008), which remained similar in subsequent models (model 3: β = −0.26, 95% CI: −0.46 to −0.06, *P* = .013; model 4: β = −0.27, 95% CI: −0.48 to −0.06, *P* = .015; and model 5: β = −0.27, 95% CI: −0.48 to −0.06; *P* = .018).

**Table 2:** Multivariate linear regression analysis of serum cotinine levels and cognitive function.

	Serum cotinine (μg/mL)	Serum cotinine β (95% CIs)		
	β (95% CIs)	<0.05 ng/mL	≥0.05 ng/mL	
Crude model	−1.66 (−2.64 to −0.69)**	1 (Ref.)		−0.69 (−1.01 to −0.37)**
Model 1	−2.29 (−3.26 to −1.33)**	1 (Ref.)		−0.72 (−0.98 to −0.46)**
Model 2	−0.89 (−1.60 to −0.17)*	1 (Ref.)		−0.29 (−0.50 to −0.09)**
Model 3	−0.80 (−1.57 to −0.02)*	1 (Ref.)		−0.26 (−0.46 to −0.06)*
Model 4	−0.84 (−1.58 to −0.10)*	1 (Ref.)		−0.27 (−0.49 to −0.06)*
Model 5	−0.85 (−1.62 to −0.08)*	1 (Ref.)		−0.27 (−0.48 to −0.06)*

Data presented are weighted β and 95% CIs.

Model 1: adjusted age, sex, and race/ethnicity.

Model 2: adjusted model 1 + PIR, partner situation, education.

Model 3: adjusted model 2 + sedentary time, general health status, BMI, and alcohol consumption.

Model 4: adjusted model 3 + depression, stroke, hypertension.

Model 5: adjusted model 4 + diabetes, heart diseases.

BMI = body mass index, CI = confidence interval, PIR = poverty-income ratio.

* *P* < .05.

** *P* < .01.

### 3.3. Sensitivity analysis and subgroup analyses

Table 3 presents the sensitivity analyses with additional adjustments for smoking status. In the complete-case analysis, the crude model showed an inverse association between categorical and cognitive function (β = −0.67, 95% CI: −0.99 to −0.35, *P* < .001), which was attenuated after additional adjustment for model 6 (β = −0.29, 95% CI: −0.51 to −0.07, *P* = .016). The continuous association also persisted in model 6 (β = −1.04, 95% CI: −1.96 to −0.12, *P* = .030). Similar patterns were observed in the imputed dataset for categorical association: (crude model: β = −0.69, 95% CI: −1.01 to −0.37, *P* < .001; model 6: β = −0.26, 95% CI: −0.47 to −0.04, *P* = .025), supporting the robustness of the association between serum cotinine and cognitive function, while the continuous association was attenuated and no longer statistically significant (β = −0.78, 95% CI: −1.63 to 0.08, *P* = .070).

**Table 3:** Sensitivity analyses with additional adjustment for smoking status.

	N	Serum cotinine (μg/mL)	Serum cotinine β (95% CIs)	
		β (95% CIs)	<0.05 ng/mL	≥0.05 ng/mL
Complete-case dataset				
Crude model	2429	−1.77 (−2.80 to −0.74)**	1 (Ref.)	−0.67 (−0.99 to −0.35)**
Model 6	2429	−1.04 (−1.96 to −0.12)*	1 (Ref.)	−0.29 (−0.51 to −0.07)*
Imputed dataset				
Crude model	2781	−1.66 (−2.64 to −0.69)**	1 (Ref.)	−0.69 (−1.01 to −0.37)**
Model 6	2781	−0.78 (−1.63 to 0.08)	1 (Ref.)	−0.26 (−0.47 to −0.04)*

Data presented are weighted β and 95% CIs.

Model 6: adjusted model 5 (age, sex and race/ethnicity, PIR, partner situation, education, sedentary time, general health status, BMI, alcohol consumption, depression, stroke, hypertension, diabetes and heart diseases) + smoking status.

BMI = body mass index, CI = confidence interval, PIR = poverty-income ratio.

* *P* < .05.

** *P* < .01.

Table 4 presents the complete-case sensitivity analysis for continuous serum cotinine across sequential models (N = 2429). The crude model showed an inverse association (β = −1.77, 95% CI: −2.80 to −0.74; *P* = .001). After adjustment for demographic factors, the association became stronger in model 1 (β = −2.43, 95% CI: −3.49 to −1.36, *P* < .001). With further adjustments, the estimates were attenuated in model 2 (β = −1.09, 95% CI: −1.92 to −0.27, *P* = .011), and remained relatively stable in subsequent models: model 3 (β = −1.05, 95% CI: −1.93 to −0.18, *P* = .021), model 4 (β = −1.08, 95% CI: −1.90 to −0.26, *P* = .014), model 5(β = −1.07, 95% CI: −1.92 to −0.22, *P* = .018), and model 6 (β = −1.04, 95% CI: −1.96 to −0.12, *P* = .030).

**Table 4:** Sensitivity analysis following the deletion of missing values based on multivariable regression analysis.

Model	β (95% CIs)	*P*
Crude model	−1.77 (−2.80 to −0.74)	.001
Model 1	−2.43 (−3.49 to −1.36)	<.001
Model 2	−1.09 (−1.92 to −0.27)	.011
Model 3	−1.05 (−1.93 to −0.18)	.021
Model 4	−1.08 (−1.90 to −0.26)	.014
Model 5	−1.07 (−1.92 to −0.22)	.018

Data presented are weighted β and 95% CIs.

Model 1: adjusted age, sex and race/ethnicity.

Model 2: adjusted model 1 + PIR, partner situation, education.

Model 3: adjusted model 2 + sedentary time, general health status, BMI, and alcohol consumption.

Model 4: adjusted model 3 + depression, stroke, hypertension.

Model 5: adjusted model 4 + diabetes, heart diseases.

BMI = body mass index, CI = confidence interval, PIR = poverty-income ratio.

**Figure 2.**
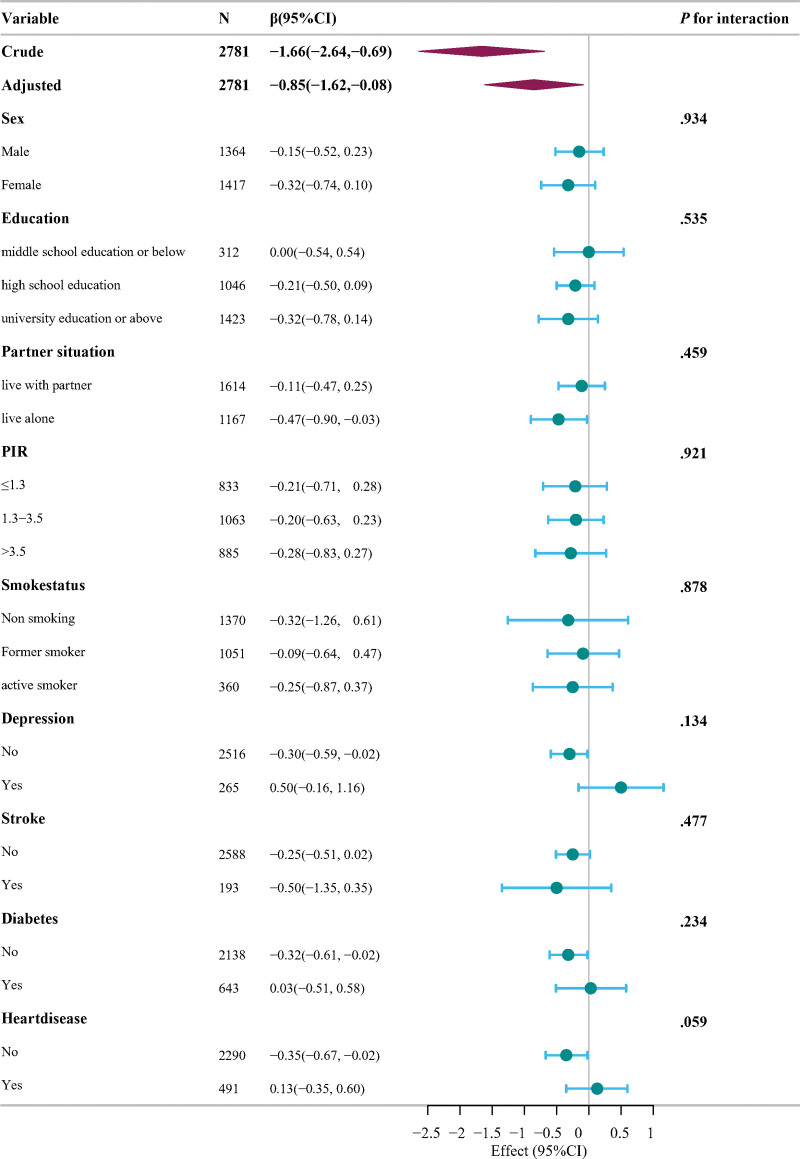
Subgroup analyses and interaction effects on the relationship between serum cotinine and cognitive function scores. CI = confidence interval, PIR = poverty-income ratio.

Figure 2 shows the subgroup analyses of the association between serum cotinine levels and cognitive function scores. An inverse association was consistently observed across all subgroups examined, with all *P* values for interactions >.05, consistent with the primary analysis.

### 3.4. Nonlinear association

**Figure 3.**
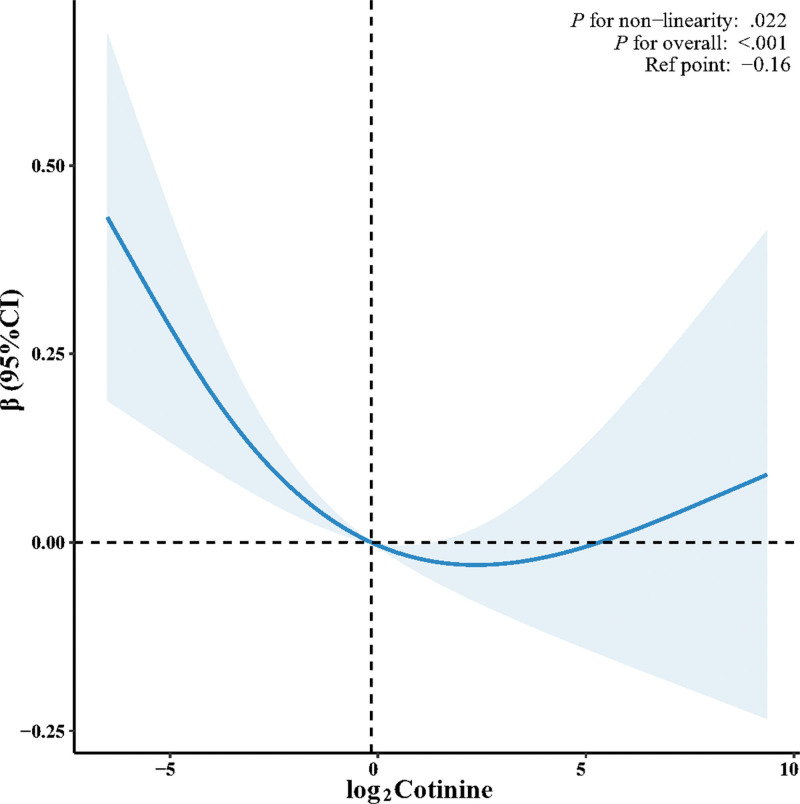
The nonlinear relationship between log_2_cotinine and cognitive function score. Adjusted for model 5. CI = confidence interval.

Figure 3 shows the nonlinear association between log_2_cotinine levels and cognitive function scores (*P* for nonlinearity = .022; *P* for overall < .001). Segmented regression identified an inflection point at log_2_cotinine = −0.16 (95% CI: −0.30 to −0.03). Below this concentration, inverse associations were observed in both the unadjusted model (crude model: β = −0.27, 95% CI: −0.40 to −0.14, *P* < .001) and the primary model (model 5: β = −0.11, 95% CI: −0.20 to −0.02, *P* = .017). Above the inflection point, the associations were not statistically significant (crude model: β = −0.05, 95% CI: −0.18 to 0.09, *P* = .491; model 5: β = −0.09, 95% CI: −0.19 to 0.01, *P* = .063).

## 4. Discussion

This cross-sectional study of US adults aged ≥60 years found an inverse, L-shaped association between serum cotinine levels and cognitive function. The inverse association was stronger below the inflection point (log_2_cotinine = −0.16) and attenuated above this threshold, with the relationship plateauing. Given its cross-sectional design, these findings represent associations rather than causal relationships. Further longitudinal studies are warranted to elucidate the temporal dynamics of this nonlinear pattern.

Data from the 2011 to 2014 cycles of this national survey were analyzed for their unique inclusion of cognitive assessments: AFT, DSST, and CERAD W-L. The AFT evaluates executive function through semantic verbal fluency (e.g., animal naming), effectively discriminating normal cognitive function from mild cognitive impairment and AD.^[19–22]^ It has minimal cultural/educational bias and has an established correlation with frontal lobe function.^[23,24]^ The DSST has demonstrated high reliability for assessing cognitive aging,^[25]^ with standardized administration ideal for population-based screening.^[26–28]^ CERAD W-L provides a cross-culturally validated memory assessment.^[24]^ Collectively, this battery profiles multidimensional cognition (executive function/processing speed/memory) with cross-cultural applicability. Although not diagnostic for domain-specific deficits,^[29]^ the standardization of these instruments and their established associations with biomarkers support their use in robust exposure-outcome analyses, as evidenced by prior high-impact research.^[30–32]^

To ensure a robust estimation of the association between cotinine and cognitive function, covariates were carefully selected based on predefined criteria. The potential association between cotinine and cognition was guided by 3 key criteria in the primary model: statistically significant univariate associations with cognitive outcomes, excluding sleep disorders and smoking status; BMI was added after covariates altered the cotinine-cognition association by >10%. Clinical relevance based on a literature review was also considered, excluding atrial fibrillation due to data limitations in the survey.^[33–36]^ This approach balanced precision with observational constraints.

Although smoking status was not included in the primary models because of its high correlation with serum cotinine and concerns about overadjustment, it remains a key potential confounder, as the mechanisms and extent of cognitive decline may differ between active smokers and nonsmokers with elevated cotinine levels from secondhand smoke (SHS) exposure. To address this, model 5 served as the primary model, whereas model 6 (with smoking adjustment) was used for the sensitivity analysis. In the complete-case analyses, both continuous and categorical associations remained statistically significant after adjustment. In the imputed dataset, the categorical association remained significant after adjusting for smoking, which was consistent with the primary analysis. However, the continuous association was attenuated and no longer reached statistical significance, even though the direction remained negative. This loss of statistical significance may reflect an insufficient sample size to detect a modest effect after stratification rather than a true absence of association. All other sensitivity analyses, including the complete-case reestimation of all models, produced results consistent with the primary findings, collectively supporting the robustness of our findings.

Previous studies have demonstrated that nicotine disrupts cognitive function throughout the lifespan. During early development, fetal nicotine exposure impairs hippocampal neurogenesis and delays cognitive maturation, reflecting developmental disruption, as evidenced by rodent models with neurobiological parallels.^[37]^ Maternal exposure during gestation and lactation further compromises offspring neurodevelopment, correlating with persistent behavioral dysfunction and an elevated risk for cognitive deficits and mood disorders.^[38]^ Adolescent neurodevelopment confers heightened limbic system vulnerability to nicotine and is characterized by dysregulated nicotinic acetylcholine receptor (nAChR) activation and persistent neuroadaptations. Human and preclinical evidence have associated chronic nicotine exposure during adolescence with sustained cognitive alterations, including attentional deficits, memory impairment, elevated impulsivity, and anxiety. Such exposure is further correlated with heightened behavioral sensitivity to substance-related rewards, potentially elevating vulnerability to subsequent dependence on substances, such as cocaine and alcohol.^[39]^ Notably, limited nicotine exposure during pediatric development correlates with preserved cognitive function in adulthood.^[40]^ In older adults, chronic nicotine exposure has been linked to accelerated cognitive decline through neurotoxic mechanisms. García-Esquinas et al^[3]^ longitudinally associated SHS exposure with an elevated risk of global cognitive impairment and working memory deficits, suggesting nicotine-accelerated neurodegeneration. Elevated tobacco-specific 4-(methylnitrosamino)-1-(3-pyridyl)-1-butanol biomarkers are inversely associated with multiple cognitive domains in older adults, including processing speed, sustained attention, and working memory efficiency.^[41]^ Collectively, these observations underscore that a reduction in SHS is a public health priority for older adult populations.

Despite consistent evidence of nicotine neurotoxicity, the cognitive-enhancing properties of nicotine and cotinine have also been described. Nicotine administration has been associated with attenuated cognitive deficits in AD and improved motor and memory function in Parkinson disease.^[42]^ Preclinical and clinical studies suggest that nicotine may be associated with enhanced performance in specific cognitive domains, particularly attention, working memory, fine motor skills, and episodic memory, through neuromodulatory mechanisms.^[43,44]^ However, the addictive potential and systemic toxicity of nicotine limit its clinical utility. Research has therefore shifted focus to its attention on cotinine, a longer-lived metabolite (half-life ~15–20 hours vs 2 hours for nicotine) associated with cognitive-enhancing effects and an improved safety profile, although its therapeutic potential remains preliminary.^[45]^ The neuroactivity of cotinine exhibits marked interspecies variation; nicotine-tolerant rats show no behavioral changes after acute dosing,^[46]^ whereas disease models show benefits, for example, memory restoration in zebrafish AD models,^[47]^ and cognitive deficit amelioration in mouse Fragile X syndrome.^[48]^ Long-term studies in animals have suggested therapeutic potential, with evidence of learning and memory enhancement in aging mice,^[49]^ reduced amyloid burden in AD models,^[50]^ arrested neurodegeneration in transgenic lines,^[51]^ and blockade of stress-induced cognitive loss via intranasal delivery.^[52]^ Conversely, in humans, data diverge, whereby acute administration (serum > 100 ng/mL) has been associated with impaired long-term memory consolidation and processing speed in nonsmokers, despite safety.^[46,53]^ However, human data on chronic cotinine exposure remain lacking, highlighting the need for rigorous studies on its safety, dosing, and target populations.

Contrary to the proposed benefits, higher cognitive function was not observed in participants exposed to nicotine in our study. The observed L-shaped association may reflect underlying biological processes; below the inflection point, the persistent inverse association could be consistent with chronic neurotoxicity (e.g., oxidative stress and vascular dysfunction). This finding aligns with that of Fu et al,^[54]^ who found that elevated serum cotinine levels were correlated with poorer cognitive performance in nonsmoking older adults, collectively consistent with tobacco-related neurotoxicity.

The inflection point identified in our study (log_2_cotinine = −0.16) corresponds to a serum cotinine concentration of approximately 0.89 ng/mL. To contextualize this value, we examined established serum cotinine thresholds for tobacco exposure.^[55]^ The broad consensus is that serum cotinine levels <0.05 ng/mL represent no detectable exposure, whereas the intermediate range of 0.05 to 10 ng/mL is generally accepted as indicative of SHS exposure. For active smoking, thresholds vary from 3 to 15 ng/mL across studies: some use 10 ng/mL,^[11]^ others use 15 ng/mL,^[56,57]^ although Pirkle et al^[58,59]^ noted that comparisons using either cutoff showed little difference. More recently, Clair et al^[60]^ proposed a lower cutoff of 3 ng/mL to better capture exposure of non-daily smokers. Within the SHS range, further stratification differs between studies. Wu et al^[11]^ classified 0.05 to 1 ng/mL as low SHS exposure and 1 to 10 ng/mL as heavy SHS exposure. Moore et al^[61]^ used 0.05 to 0.268 ng/mL for low exposure and >0.268 ng/mL for high exposure based on the median value among those with detectable cotinine. Clair et al^[60]^ categorized cotinine levels of 0.05 to 2.99 ng/mL as SHS exposure. Adding further complexity, Kermah et al^[62]^ reported that the upper limit of SHS-related cotinine varies by race/ethnicity, ranging from 0.83 ng/mL for Hispanics to 5.91 ng/mL for non-Hispanic blacks. Applying these classifications to our findings, 0.89 ng/mL falls within the SHS range and is below the active smoking thresholds. According to Wu et al,^[11]^ this value represents low-level SHS exposure. Under the Moore et al^[61]^ classification, high exposure would be considered, whereas Clair et al^[60]^ would place it within their SHS range. Despite these differing categorizations, the key observation is that 0.89 ng/mL consistently represents a level of SHS exposure below active smoking.

These findings have significant public health implications. First, it suggests that even low-level environmental tobacco exposure, well below the concentrations associated with active smoking, may be relevant to cognitive function in older adults, challenging the notion that only high-level exposure poses neurocognitive risks and highlighting the vulnerability of the aging brain to relatively low doses of tobacco-related toxins. These actions of nicotine are primarily mediated by sustained α4β2-nAChR activation.^[63]^ Specifically, chronic exposure may induce receptor desensitization and downregulation, thereby impairing cholinergic neurotransmission. High-dose exposure is associated with excessive neuroplasticity and may compromise network stability. Nicotine is also associated with oxidative stress, increased generation of reactive oxygen species that may overwhelm antioxidant defense mechanisms, and redox imbalance. This promotes neuroinflammation via tumor necrosis factor-α and interleukin release, activating microglia and damaging neurons.^[64]^ Chronic exposure may induce neuronal apoptosis (particularly in developing brains) and reduce cerebral blood flow owing to vasoconstriction. Collectively, these mechanisms, impaired cholinergic signaling, oxidative stress, neuroinflammation, apoptosis, vascular effects, and dysregulated neuroplasticity, may converge to disrupt hippocampal synaptic plasticity, directly alter short-term memory consolidation and attentional control, and ultimately compromise cognition. Second, despite these effects, a threshold effect occurs at defined nicotine concentration ranges whereby a plateau in cognitive decline occurs after saturation of nAChR in select domains.^[65]^ The location of the inflection point within the SHS range, rather than the active smoking range, suggests that the association is not uniformly dose-dependent across the entire exposure continuum. A plateau at higher concentrations primarily is consistent with saturation of neurotoxic pathways, consistent with nAChR desensitization or downregulation following chronic high-level nicotine exposure, which may limit further receptor-mediated neurotoxicity. Beyond receptor-level changes, downstream signaling pathways – including calcium influx, mitogen-activated protein kinase/extracellular signal-regulated kinase activation, and cyclic adenosine monophosphate (cAMP) response element-binding protein-mediated transcription – may also reach a ceiling, such that increasing exposure no longer amplifies neurotoxic outputs. At the cellular level, chronic exposure is linked to adaptive antioxidant responses that partially offset oxidative damage at higher concentrations, while compensatory upregulation of neurotrophic factors or anti-apoptotic pathways may buffer against progressive injury. Systemically, saturation of nicotine metabolism via cytochrome P4502A6 or blood–brain barrier transport could further contribute to the flattening of the dose–response curve at higher exposure levels. Together, these multilevel saturation effects provide a biologically plausible explanation for the nonlinear relationship observed in this older adult population. Cotinine, a primary metabolite of nicotine, exhibits substantially low pharmacological activity and lacks direct nAChR-mediated neuroactivity. Acutely elevated serum cotinine has been associated with disrupted information processing speed and executive function, evidenced by negative associations in the DSST.^[66]^ Cotinine may induce transient neuronal dysfunction via nAChR-independent mechanisms, primarily involving oxidative stress pathways.^[67]^ This oxidative dysregulation propagates via signaling cascades, suppression of cAMP/protein kinase A activity, altered cAMP response element-binding protein phosphorylation, and disruption of neuroplasticity-associated gene transcription, and ultimately contributes to cognitive decline.^[68]^ At elevated concentrations, cotinine activates endogenous antioxidant pathways that partially offset neurocognitive toxicity and correlate with specific impairments in processing speed and executive function.^[69]^ Serum cotinine concentrations can quantify environmental tobacco exposure. Lower levels are associated with reduced xenobiotic tolerance and greater susceptibility to cognitive impairment, which is consistent with sporadic exposure. Conversely, chronic exposure is associated with adaptive tolerance and attenuated cognitive differences.^[70]^ This interplay of physiological compensation and interindividual tolerance heterogeneity may contribute to the observed cognitive plateau and provide insight into the potential role of cotinine in cognitive function. As an alternative consideration, the observed plateau may be partly attributable to the “healthy smoker effect,” which is a form of survivorship bias whereby individuals who continue smoking into older age represent a subgroup with greater physiological resilience, potentially attenuating the association at higher concentrations. Differential measurement errors across exposure levels are another possibility, although less likely, given the reliability of cotinine as a biomarker. In summary, nicotine and cotinine exhibit context-dependent associations with cognitive function through complex biological pathways. From a public health perspective, these findings highlight that reducing even modest SHS exposure may be relevant to the cognitive health of older populations.

### 4.1. Study strengths

This study has several methodological strengths. The use of serum cotinine as a biomarker for tobacco exposure in a nationally representative sample of US adults aged ≥60 years provides novel insights that complement prior research. Weighted analyses enhanced national representativeness and comprehensive confounder adjustment, and subgroup analyses reinforced the robustness of the observed associations. Missing data were addressed through imputation with sensitivity analyses excluding the imputed values, which yielded consistent results. Collectively, the approaches used in this study, including biomarker-based exposure assessment, weighted sampling, rigorous confounder with control subgroup validation, and missing data handling, strengthened the validity of the findings.

### 4.2. Limitations and future research directions

Despite these strengths, this study has some limitations. First, the cross-sectional design precludes any determination of causal inference; reverse causality cannot be ruled out. Individuals with poorer cognitive function may be more likely to smoke or have higher SHS exposure. Second, residual confounding may persist owing to unmeasured factors, such as cumulative pack-years or cessation duration, among former smokers. Third, the absence of atrial fibrillation data constrains the interpretation, although the adjustment for cardiovascular conditions partially mitigates this concern. Fourth, missing values may reduce precision; however, consistent effect sizes across the imputed and complete-case analyses support robustness. Fifth, the limitations of the cognitive measures included domain non-specificity, ceiling effects in highly educated subgroups, and limited sensitivity to executive dysfunction, which were addressed through adjustment for education level and available executive function assessments. Sixth, the identified threshold reflects a population-level statistical trend rather than a biological boundary, and the cross-sectional design does not allow us to distinguish between acute and chronic effects. Longitudinal studies and Mendelian randomization analyses are warranted to further explore this association.

## 5. Conclusions

This cross-sectional study identified an inverse L-shaped association between serum cotinine levels and cognitive function in older US adults. These findings contribute to the growing evidence supporting tobacco-related neurocognitive effects and generate further hypotheses for future research. Further investigations are required to elucidate the mechanisms underlying the observed threshold effect.

## Acknowledgments

We gratefully acknowledge Jie Liu of the Department of Vascular and Endovascular Surgery, Chinese PLA General Hospital, for his contribution to statistical support, study design consultations, and comments regarding the manuscript, and Qilin Yang of the Department of Critical Care Medicine, The Second Affiliated Hospital of Guangzhou Medical University, for his feedback. We also thank all participants in the survey and the research team for collecting and sharing the data.

## Author contributions

**Data curation:** Jieqiong Zhang, Guanxun Zhang, Ciai Yan.

**Formal analysis:** Jieqiong Zhang.

**Methodology:** Jieqiong Zhang, Zhixin Sun, Weiping Cheng.

**Software:** Jieqiong Zhang, Guanxun Zhang, Ciai Yan.

**Resources:** Guanxun Zhang.

**Visualization:** Guanxun Zhang, Ciai Yan, Guangyu Cheng.

**Conceptualization:** Weiping Cheng.

**Writing – original draft:** Jieqiong Zhang, Guanxun Zhang, Ciai Yan, Zhixin Sun.

**Writing – review & editing:** Jieqiong Zhang, Guangyu Cheng, Weiping Cheng.


