# Supplementary material for: Association between serum cotinine levels and cognitive function in Americans aged 60 and older: A cross-sectional study
Source: Medicine (Baltimore). 2026 May 22;105(21):e48784. doi: 10.1097/MD.0000000000048784 (PMC13200992; doi:10.1097/MD.0000000000048784)
Supplement: Supplementary file 1 [file medi-105-e48784-s001.docx]

**Supplementary Table 1 Univariate linear regression analysis of variates and Cognitive Function in Americans Aged ≥60 years.**

| **Variables** | **β** | **95%CI** | ***P* value** |  |
| --- | --- | --- | --- | --- |
|  |  |  |  |  |
| **Cotinine(ug/mL)** | -1.66 | -2.64, -0.69 | .002 |  |
| **Age (years)** | -0.15 | -0.16, -0.14 | <.001 |  |
| **Sex** |  |  |  |  |
| Male | 1(Ref) |  |  |  |
| Female | 0.39 | 0.20, 0.58 | <.001 |  |
| **Race/Ethnicity** |  |  |  |  |
| Non-Hispanic White | 1(Ref) |  |  |  |
| Non-Hispanic Black | -1.66 | -2.06, -1.26 | <.001 |  |
| Mexican American | -1.46 | -1.96, -0.96 | <.001 |  |
| Other Hispanic or Multi-Racial | -1.35 | -1.83, -0.87 | <.001 |  |
| *P* for trend |  |  | <.001 |  |
| **Education** |  |  |  |  |
| middle school education or below | 1(Ref) |  |  |  |
| high school education | 1.83 | 1.30, 2.37 | <.001 |  |
| university education or above | 3.46 | 2.99, 3.94 | <.001 |  |
| *P* for trend |  |  | <.001 |  |
| **Partner situation** |  |  |  |  |
| live with partner | 1(Ref) |  |  |  |
| live alone | -0.65 | -0.91, -0.39 | <.001 |  |
| **PIR** |  |  |  |  |
| ≤1.3 | 1(Ref) |  |  |  |
| 1.3-3.5 | 1.04 | 0.75, 1.33 | <.001 |  |
| ＞3.5 | 2.36 | 2.02, 2.70 | <.001 |  |
| *P* for trend |  |  | <.001 |  |
| **Sedentary time(hours)** | -0.01 | -0.02, -0.01 | <.001 |  |
| **General health status** |  |  |  |  |
| Good | 1(Ref) |  |  |  |
| Fair | -1.59 | -1.84, -1.34 | <.001 |  |
| Poor | -2.02 | -2.83, -1.21 | <.001 |  |
| *P* for trend |  |  | <.001 |  |
| **BMI (kg/m^2^)** | 0.01 | -0.02, 0.03 | .618 |  |
| **Smoking status** |  |  |  |  |
| Non-smoking | 1(Ref) |  |  |  |
| Former smoker | -0.25 | -0.57, 0.07 | .12 |  |
| active smoker | -0.49 | -0.87, -0.10 | .015 |  |
| *P* for trend |  |  | .017 |  |
| **Alcohol consumption** |  |  |  |  |
| No | 1(Ref) |  |  |  |
| Yes | 0.87 | 0.59, 1.15 | <.001 |  |
| **Sleep disorders** |  |  |  |  |
| No | 1(Ref) |  |  |  |
| Yes | 0.07 | -0.30, 0.45 | .685 |  |
| **Depression** |  |  |  |  |
| No | 1(Ref) |  |  |  |
| Yes | -1.15 | -1.65, -0.65 | <.001 |  |
| **Hypertension** |  |  |  |  |
| No | 1(Ref) |  |  |  |
| Yes | -1 | -1.29, -0.71 | <.001 |  |
| **Heart disease** |  |  |  |  |
| No | 1(Ref) |  |  |  |
| Yes | -1.02 | -1.33, -0.70 | <.001 |  |
| **Diabetes** |  |  |  |  |
| No | 1(Ref) |  |  |  |
| Yes | -0.96 | -1.33, -0.59 | <.001 |  |
| **Stroke** |  |  |  |  |
| No | 1(Ref) |  |  |  |
| Yes | -1.36 | -1.84, -0.88 | <.001 |  |

Notes: Weighted analyses were applied to all estimates. PIR: Poverty Income Ratio; BMI, body mass index.
